# Whole-Body Prolyl Hydroxylase Domain (PHD) 3 Deficiency Increased Plasma Lipids and Hematocrit Without Impacting Plaque Size in Low-Density Lipoprotein Receptor Knockout Mice

**DOI:** 10.3389/fcell.2021.664258

**Published:** 2021-05-14

**Authors:** Jasper A. F. Demandt, Kim van Kuijk, Thomas L. Theelen, Elke Marsch, Sean P. Heffron, Edward A. Fisher, Peter Carmeliet, Erik A. L. Biessen, Judith C. Sluimer

**Affiliations:** ^1^Department of Pathology, Cardiovascular Research Institute Maastricht (CARIM), Maastricht University Medical Center (MUMC), Maastricht, Netherlands; ^2^Institute of Experimental Medicine and Systems Biology, RWTH Aachen University, Aachen, Germany; ^3^Center for the Prevention of Cardiovascular Disease, Department of Medicine, Grossman School of Medicine, New York University, New York, NY, United States; ^4^Laboratory of Angiogenesis and Vascular Metabolism, Department of Oncology, KU Leuven, Leuven, Belgium; ^5^Laboratory of Angiogenesis and Vascular Metabolism, VIB Center for Cancer Biology, Leuven, Belgium; ^6^Institute for Molecular Cardiovascular Research, RWTH Aachen University, Aachen, Germany; ^7^BHF Centre for Cardiovascular Sciences (CVS), University of Edinburgh, Edinburgh, United Kingdom

**Keywords:** atherosclerosis, triglycerides, prolyl hydroxylase domain protein, hematocrit, hypoxia

## Abstract

**Background and aims:** Atherosclerosis is an important cause of clinical cardiovascular events. Atherosclerotic plaques are hypoxic, and reoxygenation improves plaque phenotype. Central players in hypoxia are hypoxia inducible factors (HIF) and their regulators, HIF-prolyl hydroxylase (PHD) isoforms 1, 2, and 3. PHD inhibitors, targeting all three isoforms, are used to alleviate anemia in chronic kidney disease. Likewise, whole-body PHD1 and PHD2ko ameliorate hypercholesterolemia and atherogenesis. As the effect of whole-body PHD3 is unknown, we investigated the effects of germline whole-body PHD3ko on atherosclerosis.

**Approach and Results:** To initiate hypercholesterolemia and atherosclerosis low-density lipoprotein receptor knockout (LDLrko) and PHD3/LDLr double knockout (PHD3dko), mice were fed a high-cholesterol diet. Atherosclerosis and hypoxia marker pimonidazole were analyzed in aortic roots and brachiocephalic arteries. In contrast to earlier reports on PHD1- and PHD2-deficient mice, a small elevation in the body weight and an increase in the plasma cholesterol and triglyceride levels were observed after 10 weeks of diet. Dyslipidemia might be explained by an increase in hepatic mRNA expression of Cyp7a1 and fatty acid synthase, while lipid efflux of PHD3dko macrophages was comparable to controls. Despite dyslipidemia, plaque size, hypoxia, and phenotype were not altered in the aortic root or in the brachiocephalic artery of PHD3dko mice. Additionally, PHD3dko mice showed enhanced blood hematocrit levels, but no changes in circulating, splenic or lymphoid immune cell subsets.

**Conclusion:** Here, we report that whole-body PHD3dko instigated an unfavorable lipid profile and increased hematocrit, in contrast to other PHD isoforms, yet without altering atherosclerotic plaque development.

## Introduction

Atherosclerosis frequently stands at the origin of cardiovascular disease (CVD). Next to the major risk factors like inflammation (Tabas and Bornfeldt, 2016) and dyslipidemia (Plump et al., 1992; Zhang et al., 1992; Ishibashi et al., 1994), hypoxia contributes to the progression of atherosclerotic plaques (Marsch et al., 2014). Critical in this hypoxic signaling cascade are the hypoxia inducible factor (HIF) family members HIF1α and HIF2α. This master transcription factor family steers multiple cellular adaptations, in order to maintain normal cellular functions during hypoxic conditions. These adaptations include metabolic changes, affecting glucose (Samanta and Semenza, 2018) and lipid (Mylonis et al., 2019) metabolism. Also, inflammatory pathways are affected by HIF signaling, and multiple studies have shown that altered HIF1α signaling interferes with atherosclerotic plaque development (Ben-Shoshan et al., 2009; Christoph et al., 2014; Akhtar et al., 2015; Aarup et al., 2016; Jain et al., 2018). The HIFα subunits are constitutively expressed, but can only exert their functions upon translocation and subsequent dimerization with HIF1β in the nucleus. HIF activity is predominantly regulated in an oxygen-dependent manner by the egl-9 family hypoxia inducible factors (EGLN) family, hereafter referred to as HIF-prolyl hydroxylase enzymes PHD1, 2, and 3 (EGLN2, 1, and 3) (Kaelin and Ratcliffe, 2008). Each of these Fe^2+^ and 2-oxoglutarate-dependent dioxygenases shows a different intracellular localization and affinity for HIF1α and HIF2α (Berra et al., 2003; Appelhoff et al., 2004). Oxygen is used by the PHDs to hydroxylate a proline residue of the HIF1α and HIF2α subunits. Hydroxylated HIF is ubiquitinated by Von Hippel-Lindau proteins, rendering it a target for proteasomal degradation (Appelhoff et al., 2004). PHD2 is considered the most important isoform *in vivo*, as homozygous PHD2 knockout (ko) embryos die between day 12.5 and 14.5 of gestation (Takeda et al., 2006). PHD3 is the most strongly induced isoform in reaction to hypoxia, followed by PHD2 (Appelhoff et al., 2004; Aprelikova et al., 2004). This makes PHD3 an interesting target for further investigation in the hypoxic atherosclerotic plaque.

Recently, our group showed that whole-body PHD1 deficiency in atherosclerosis-prone low-density lipoprotein receptor knockout (LDLR-ko) mice reduced atherosclerotic plaque size and necrotic plaque content as a result of protective effects on extrahepatic lipid metabolism and macrophage oxygen consumption (Marsch et al., 2016). Another group reported reduced plasma lipid levels due to hepatic effects and protective autoantibodies against oxidized lipids in hypomorphic PHD2/LDLR^C699Y^ mutant mice (Rahtu-Korpela et al., 2016). This resulted in ameliorated plaque size, indicating the importance of PHD proteins in metabolism and subsequent plaque development. In line, PHD inhibitors, targeting all three isoforms to alleviate anemia in chronic kidney disease, also ameliorated lipid metabolism in mice and humans, and atherogenesis in mice (Rahtu-Korpela et al., 2016; Chen et al., 2019a, b). Considering that CKD patients are at risk of CVD, plaques are hypoxic, PHDs can interfere with metabolism, and PHD3 is strongly upregulated during hypoxia. PHD3 is an attractive target to study in the context of atherosclerosis. Hence, we set out to study the effects of whole-body PHD3ko on plaque development.

## Materials and Methods

### Animals and Atherosclerosis Model

All mouse experiments were approved by the regulatory authority of Maastricht University Medical Centre and performed in compliance with the guidelines described in the Directive 2010/63/EU of the European Parliament. All mice were bred at least nine generations on C57/JBl6 background, and male low-density lipoprotein receptor (LDLr) knockout (ko) mice were obtained from an in-house breeding colony, originating from Charles River (Wilmington, MA, United States) and refreshed every 10 generations to avoid genetic drift. All animals were housed in individually ventilated cages (GM500, Tecniplast) in groups of up to five animals per cage, with bedding (corncob, Tecnilab-BMI) and cage enrichment. Cages were changed weekly, reducing handling of the mice to once per week during non-intervention periods. Male PHD3 and LDLr double knockout (PHD3dko) and LDLrko littermates (*n* = 14 and 22, respectively, 11 weeks old) were fed a high-cholesterol diet (HCD) *ad libitum* (0.25% cholesterol, SDS 824171) for 10 weeks.

### Atherosclerosis Quantification and Immunohistochemistry

One hour prior to sacrifice, all mice were intraperitoneally (i.p.) injected with the hypoxia-specific marker pimonidazole (100 mg/kg, hypoxyprobe Omni HP3 kit, Hypoxyprobe Inc., Burlington, MA, United States). Mice were euthanized with a pentobarbital overdose (100 mg/kg i.p.), and blood was withdrawn via the right ventricle for flow cytometry, absolute white and red blood cell counts (Coulter Ac.T diff, Beckman Coulter, United States), and total cholesterol analysis. Mice were perfused via the left cardiac ventricle with PBS containing sodium nitroprusside (0.1 mg/ml; Sigma-Aldrich, Seelze, Germany). Aortic root and all organs were subsequently excised and fixed in 1% PFA overnight, processed, and paraffin-embedded.

Aortic roots and arches were serially sectioned and stained with hematoxylin and eosin (H&E, Sigma) for plaque area and lipid core content quantification. Plaque stage was quantified on plaque characteristics such as foam cells (early), fibrous cap (intermediate), and necrotic core (advanced). Five consecutive H&E sections at 20 μm intervals were analyzed blindly using computerized morphometry (Leica QWin V3, Cambridge, United Kingdom) and averaged per mouse. Sections within this 100 μm interval were used for the remaining immunohistochemical stainings. If appropriate, antigen retrieval was performed at pH6 (Dako REAL target retrieval, Dako). Atherosclerotic plaques were characterized for macrophage content (MAC3 + area/total area, BD Cat# 553322, RRID:AB_394780). Hypoxia was detected in the aortic roots, using a rabbit polyclonal antibody (clone 2627, Cat# HP MAb-1, RRID:AB_2801307) directed against pimonidazole derivates, formed *in vivo* specifically in hypoxic but living cells (% pimonidazole/total plaque area). Liver inflammation was quantified as percentage CD45 + cells/total visible area (BD Cat# 553076, RRID:AB_394606).

### Total and Hepatic Cholesterol and Triglycerides

Plasma was separated by centrifugation and stored at –80°C until further use. Standard enzymatic techniques were used to assess plasma cholesterol (cholesterol FS’10; Ref: 1 1300 99 10 021; Diagnostic Systems GmbH, Holzheim, Germany) and plasma triglycerides (TG) (FS5’ Ecoline REF 157609990314; DiaSys—Diagnostic Systems GmbH, Holzheim, Germany) automated on the Cobas Fara centrifugal analyzer (Roche). For hepatic cholesterol and TG content, livers were homogenized in SET buffer (250 mM sucrose, 2 mM EDTA, 10 mM Tris, pH 6.8). Upon two freeze-thaw cycles and suction of the homogenate through an insulin syringe, TG and cholesterol were measured in the homogenates using the kits described above. Cholesterol and TG levels were corrected for protein content assessed in the same homogenate using a BCA kit (Thermo Fisher Scientific, Cat. No. 23227).

### Flow Cytometry and Blood Variables

Circulating cells were isolated from whole blood and analyzed using flow cytometry (*n* = 10 per group). Blood was subjected to erythrocyte lysis. The following specific antibodies were used to detect leukocyte subsets: leukocytes (CD45 +, BioLegend Cat# 103129, RRID:AB_893343), T cells (CD3ε +, NK1-1-; Miltenyi, eBioscience Cat# 12-5941-82, RRID:AB_466050), T helper cells (CD4 +, BD Cat# 560246, RRID:AB_1645236), cytotoxic T cells (CD8a +, BD Cat# 560776, RRID:AB_1937317), effector T cells (CD44high CD62low; BD Cat# 560568, RRID:AB_1727481, eBioscience Cat# 25-0621-82, RRID:AB_469633, resp.), B cells (B220 +; BD Cat# 561226, RRID:AB_10563910), NK cells (NK1-1 +, BD Cat# 557391, RRID:AB_396674), eosinophils (SiglecF +; BD Cat# 562681, RRID:AB_2722581), granulocytes (CD11bhigh Ly6Ghigh; BD Cat# 552850, RRID:AB_394491, eBioscience Cat# 12-5931-82, RRID:AB_466045), and monocytes (CD11bhigh Ly6Glow Ly6Chigh/intermediate/low; Miltenyi Cat# 130-093-136, RRID:AB_871571). Data were acquired using a FACS Canto II and analyzed with FACS diva software (BD). For erythropoietic variable analysis, whole blood was diluted 1:10 in Hepes buffer, pH 7.45 (10 mM Hepes, 136 mM NaCl, 2.7 mM KCl, 2 mM MgCl2, 0.1% glucose, 0.1% BSA) and subsequently measured on the XP3000 Sysmex analyzer (Sysmex, Chuo-ku Kobe, Japan).

### Cell Culture

Bone marrow was isolated, and cells were cultured for 7 days in RPMI-1640 (Gibco with Glutamax, 2 g/L glucose) supplemented with 10% FCS, 100 U/ml penicillin–streptomycin, and 15% L929-conditioned medium to generate bone marrow-derived macrophages (BMDM).

### Real-Time Quantitative PCR

Cells were cultured accordingly, and RNA was isolated and produced as described (Sluimer et al., 2007). qPCR analyses were performed from 10 ng cDNA using SYBR green (Bio-Rad), and gene-specific primer sequences are available on request (Eurogentec, Liege, Belgium). One housekeeping gene (18S) was used to correct for different mRNA quantities between samples.

### Insulin ELISA

Insulin was measured in plasma of LDLrko and PHD3dko mice based on the manufacturers’ protocol (Mercodia, 10-1247-01).

### Bone Marrow-Derived Macrophage Cholesterol Efflux

PHD3dko and LDLrko BMDMs were loaded overnight with acetylated LDL (50 μg/ml, Alfa Aesar J65029) and 1 uCi/mL H^3^-cholesterol with or without 2 ug/mL ACAT inhibitor (Sandoz 58-035, Sigma). To increase ABCA1 expression, BMDMs were incubated with 0.3 mmol/L cAMP (Sigma-Aldrich, C3912) for 6 h and were then exposed to purified human HDL 50 ug/mL (Alfa Aesar, Haverhill, MA, United States) in media for 4 h. After these 4 h, the HDL in media was removed, macrophages were lysed, and scintillation counting was used to quantify the amount of ^3^H-cholesterol in each compartment. We calculated the cholesterol efflux capacity as follows: [microcuries ^3^H in media containing human HDL/ApoA1—microcuries ^3^H in serum-free media/(total microcuries ^3^H in media and cell lysate)] x 100%. This value represents the total cholesterol efflux capacity. To be able to determine ABCA/G1-mediated cholesterol efflux capacity, we used the same protocol on BMDMs which are not treated with cAMP prior to HDL exposure. The difference between cAMP-induced (global) and non-cAMP-induced cholesterol efflux represents cAMP inducible or ABCA/G1-mediated cholesterol efflux.

### Statistical Analysis

All data are presented as mean ± SEM. All variables were analyzed using independent sample tests and were tested for outliers and normal distribution using Grubs test and Shapiro–Wilk normality test, respectively. Variables with two groups were compared with student’s *t*-test or Mann–Whitney rank-sum test. In case of more than two groups, variables were analyzed using one-way ANOVA followed by Bonferroni’s multiple comparison test. A *p*-value of <0.05 was considered significant (^∗^*p* < 0.05, ^∗∗^*p* < 0.01, ^∗∗∗^*p* < 0.001).

## Results

### Vascular Cell Types Expressed PHD3 and PHD3 Deficiency Enhanced Red Blood Cell Count

**FIGURE 1:**
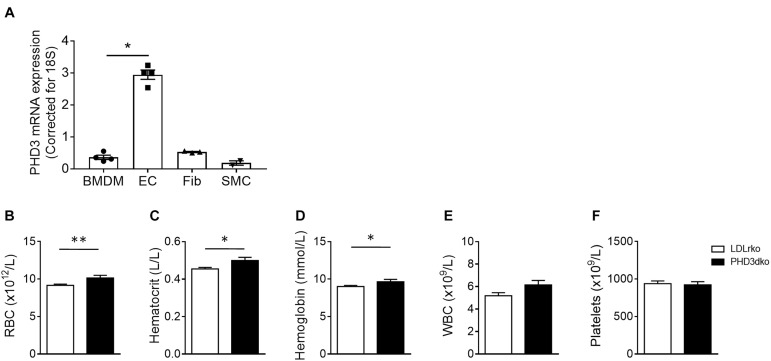
All vascular cells expressed PHD3 and increased Hb and Ht confirmed functional PHD3ko. **(A)** PHD3 mRNA expression in vascular cells involved in atherosclerosis *in vitro* (*n* = 4 replicates per group). Expression is depicted as relative expression, corrected for 18S. **(B–F)** Hematopoietic variables of LDLrko (*n* = 21) and PHD3dko (*n* = 14) mice measured from blood drawn upon sacrifice. **(B)** Red blood cells (RBC), **(C)** Hematocrit, **(D)** Hemoglobin, **(E)** White blood cells (WBC), and **(F)** Platelets. BMDM, bone marrow derived macrophage; SMC, smooth muscle cell; Fib, 3T3 fibroblast; EC, mouse cardiac endothelial cell. All results show mean ± SEM. ^∗^*p* < 0.05, ^∗∗^*p* < 0.01.

PHD3 was expressed by all major cell types involved in atherosclerosis (Figure 1A) and its deletion might hence change the course of atherogenesis. PHD3 is an important regulator of HIF1α and HIF2α activities, the latter being the main driver of EPO production (Warnecke et al., 2004; Scortegagna et al., 2005; Kapitsinou et al., 2010). As expected, plasma erythrocyte, hematocrit, and hemoglobin were enhanced in PHD3dko compared to controls at the moment of sacrifice (Figures 1B–F). White blood cell count and platelets were not significantly different. These results confirm PHD3ko in our mice at functional level.

### Whole-Body PHD3dko Increased Murine Body Weight and Circulating Lipids

**FIGURE 2:**
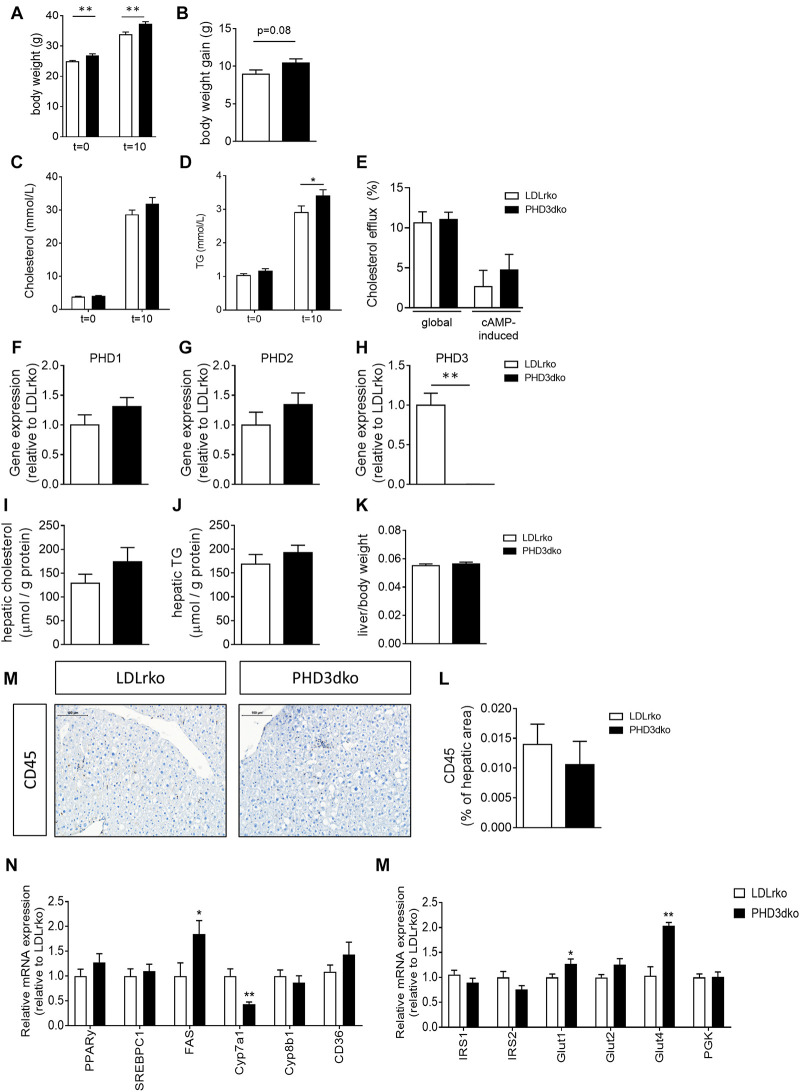
PHD3dko mice showed increased bodyweight and circulating lipids. **(A)** Body weight of PHD3dko and LDLrko mice at start and end of the diet. **(B)** Body weight gain measured after 10 weeks of diet. **(C,D)** Plasma cholesterol and TG levels at start of diet and 10 weeks of diet. **(E)** Global and cAMP-induced cholesterol efflux capacity of LDLrko and PHD3dko BMDMs. **(F)** Gene expression of PHD1, **(G)** PHD2, and **(H)** PHD3 in whole liver tissue of LDLrko and PHD3dko mice (*n* = 5/group). Expression is relative to LDLrko expression levels of respective gene of interest. **(I,J)** Hepatic cholesterol (PHD3dko *n* = 20, LDLrko *n* = 14) and TG (PHD3dko *n* = 20, LDLrko *n* = 13) content of mouse livers corrected for total amount of hepatic protein. **(K)** Liver weight corrected for total body weight. **(L)** Representative microphotographs of CD45 stained sections of liver. Scalebar represents 100 μm. **(M)** Hepatic inflammation as measured by CD45 positive cells per visible hepatic area (PHD3dko *n* = 21, LDLrko *n* = 14). **(N)** Gene expression of lipid and glucose homeostasis related genes in livers of PHD3dko mice. Expression depicted relative to LDLrko expression levels of respective gene of interest (LDLrko *N* = 9, PHD3dko *N* = 7). PPARγ, peroxisome proliferator-activated receptor gamma; SREBPc1, sterol regulatory element-binding protein; FAS, fatty acid synthase; CYP7a1/8b1, cytochrome P450 7a1/8b1; CD36, cluster of differentiation 36; Glut1/2/4, glucose transporter 1, 2, or 4; IRS1/2, insulin receptor substrate 1/2. Sample size *n* = 14 for PHD3dko and *n* = 22 for LDLrko groups unless otherwise stated. All results show mean ± SEM. *p < 0.05, **p < 0.01.

Before the start of the high-cholesterol diet (*t* = 0) and prior to sacrifice (*t* = 10), body weight was measured. Unexpectedly, PHD3dko mice showed enhanced body weight both before and after the start of the diet (Figure 2A). Enhanced body weight at the end of the diet might not only be attributed to enhanced mass prior to the diet, as PHD3dko mice also showed a trend toward more weight gain during the study (*p* = 0.08) (Figure 2B). Increased body mass was accompanied by a small increase in circulating total cholesterol (+7.08%) and significantly elevated TG levels (+17.9%) after 10 weeks of diet, while baseline measurements showed no difference (Figures 2C,D). Lipid homeostasis is strongly influenced by hepatic uptake, synthesis, and biliary excretion of cholesterol and to a minor extent also by reversed cholesterol transport by macrophages (Afonso et al., 2018; Westerterp et al., 2018). Reversed cholesterol transport probably does not explain circulating lipid levels, as *in vitro* BMDM cholesterol efflux capacity was unchanged (Figure 2E). Hence, the hepatic phenotype was studied. Importantly, PHD3 was successfully knocked out in the livers of PHD3dko mice, without compensatory upregulation of other PHD isoforms (Figures 2F–H). Liver lipid content, weight, and inflammation were not different between both groups (Figures 2I–L). mRNA expression of multiple lipid homeostasis-related genes in PHD3dko and control livers revealed significant changes in fatty acid synthase (FAS) and Cyp7a1 (Figure 2N). As alterations in these genes lead to an imbalance in lipid profiles, this could be the explanation for our blood lipid phenotype. Next to lipid homeostasis, we also investigated genes related to glucose and insulin signaling in PHD3dko and control livers (Figure 2M). We observed an increase in insulin-independent and insulin-dependent glucose transporters, Glut1/Glut2 and Glut4, respectively. Despite the enhanced expression, we were unable to show an increase in insulin levels in the plasma (Supplementary Figure 1, data not shown).

### PHD3dko Did Not Affect Plaque Size Despite Inducing Hyperlipidemia

**FIGURE 3:**
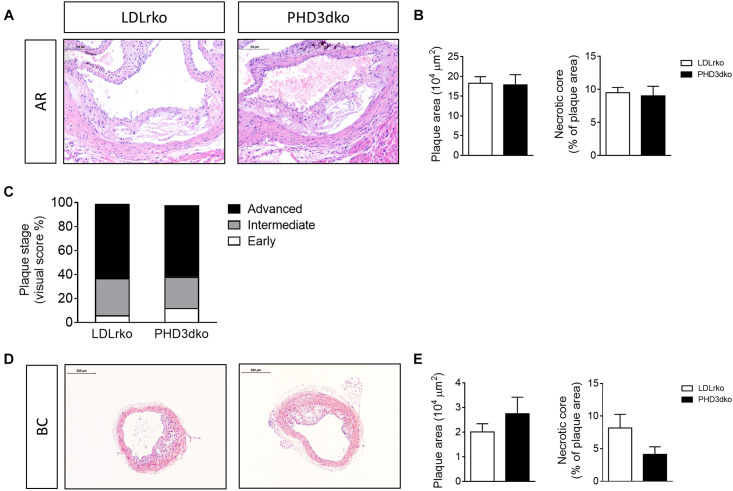
PHD3dko did not change plaque size or necrotic content. **(A)** Representative microphotographs of H&E stained sections of aortic root (AR) (PHD3dko *n* = 18, LDLrko *n* = 10) and **(B)** quantification of plaque size and necrotic plaque content. **(C)** Plaque stage represented as early, intermediate, or advanced. **(D)** Representative microphotographs of H&E stained sections of brachiocephalic artery (BC) (PHD3dko *n* = 21, LDLrko *n* = 13), and **(E)** quantification of plaque size and necrotic plaque content. Scalebar represents 100 μm in AR and 200 μm in BC photographs.

**FIGURE 4:**
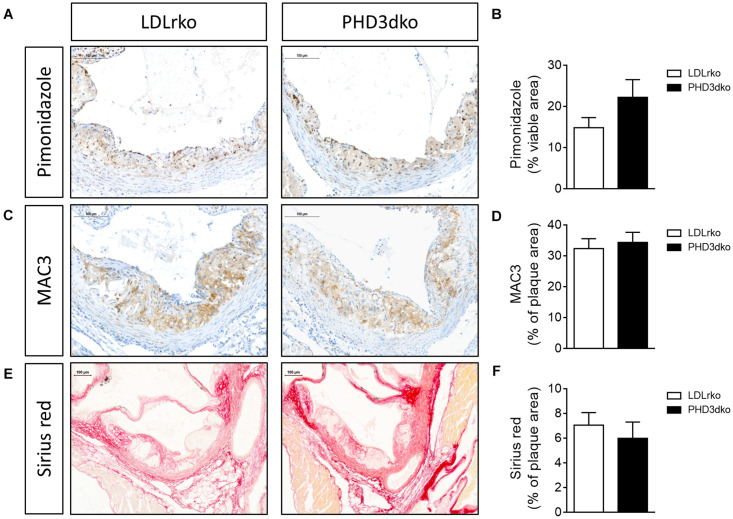
PHD3dko did not affect plaque hypoxia, macrophage or collagen content. **(A)** Representative microphotographs of aortic root sections stained for hypoxia content (pimonidazole), **(B)** quantification (PHD3dko *n* = 18, LDLrko *n* = 10), **(C)** macrophage content (MAC3), and **(D)** quantification (PHD3dko *n* = 16, LDLrko *n* = 10), **(E)** collagen content (Picro sirius red), and **(F)** quantification (PHD3dko *n* = 18, LDLrko *n* = 11). Scalebar represents 100 μm.

Given the increased plasma lipid levels, albeit modest, we expected a small aggravation of atherogenesis in PHD3dko mice. However, the plaque size and the necrotic plaque content in the aortic root were unchanged compared to controls (Figures 3A,B). We reasoned that we could not find an effect on atherogenesis, as plaques had progressed to a fulminant stage, where differences might not be observed anymore. This was confirmed in the plaque stage classification, illustrating that the majority of plaques are advanced, irrespective of PHD3 deletion (Figure 3C). Hence, we also assessed plaque size at a second location, the brachiocephalic artery, where plaque development starts at a later stage (Nakashima et al., 1994). In line with findings in the aortic root, no differences were observed in plaque size or necrotic core content of the brachiocephalic artery, with less advanced lesions than the aortic root (Figures 3D,E). Accordingly, plaque hypoxia, macrophage, and collagen content in the aortic root were not significantly different between both groups (Figures 4A–F). Since whole-body PHD1ko on an LDLrko background led to changes in circulating immune cell subsets, we checked whether PHD3dko could lead to similar effects. However, in line with unchanged macrophage content in the plaque, PHD3dko did not lead to changes in circulating leukocyte and lymphocyte subsets between both groups (Supplementary Figures 2A–L).

## Discussion

In this study, we set out to investigate the effects of whole-body PHD3 deficiency on atherosclerotic plaque development. Despite enhanced lipid and hematocrit levels in whole-body PHD3dko mice, we did not observe an effect on atherogenesis.

Our group and others already showed the significance of PHD proteins in regulating total circulating cholesterol and/or triglyceride levels, which are important contributors to atherogenesis. In our prior study, whole-body PHD1 deficiency was seen to decrease plasma cholesterol and TG levels by ∼30%, subsequently decreasing plaque size and necrotic plaque content in these mice (Marsch et al., 2016). Comparable findings were apparent in hypomorphic PHD2ko mice, with reduced plaque size and advantageous lipid homeostasis (Rahtu-Korpela et al., 2014, 2016). As opposed to PHD1 and PHD2 deficiency, here we report an increase in plasma lipids in PHD3dko mice, albeit only by 10–18%. This may simply be insufficient to alter atherogenesis. The clinical relevance of this moderate increase in cholesterol levels upon high-cholesterol diet feeding is unclear. In a clinical trial testing Vadadustat, a moderately selective PHD3 inhibitor, no changes were found in circulating cholesterol or triglyceride levels (Martin et al., 2017). However, Vadadustat also inhibits PHD2, albeit to a lesser extent. Considering that several PHD2 selective inhibitors reduced lipid levels in humans, the latter study might suggest that potentially harmful effects of PHD3 inhibition may have been masked by the co-inhibition of PHD2. This highlights that PHD3 influences metabolism in an essentially different way than the PHD1 and PHD2 isoforms.

Why whole-body PHD3dko acts hyperlipidemic instead of hypolipidemic, like PHD1 and PHD2, remains elusive. PHD3 deficiency may have led to HIF2α-mediated changes in lipid metabolism, as HIF2α is known to participate in lipid homeostasis. In hepatic PHD3ko mice, glucose tolerance and insulin sensitivity were increased in a HIF2α, but not HIF1α, dependent way by upregulating insulin receptor substrate 2 expression (Taniguchi et al., 2013). Increased insulin sensitivity is, however, expected to promote an advantageous lipid profile, which was not observed in our whole-body PHD3dko model. Moreover, a lack of increase in the plasma insulin and in the hepatic expression of insulin responsive genes suggests that insulin sensitivity was unchanged in whole-body PHD3dko mice. As high-fat diet with 60%kcal from fat, rather than high-cholesterol diet (21%kcal fat), would be needed to induce overt glucose intolerance, the current high-cholesterol study is not suitable to yield conclusions on glucose and/or insulin intolerance. Alternatively, CYP7a1 may underlie the observed effect. Interestingly, liver-specific pVHL knockout mice displayed hypercholesterolemia, an effect that was HIF2α dependent, and presumably involved an inhibition of Cyp7a1-mediated conversion of cholesterol into bile acids (Ramakrishnan et al., 2014). Of note, lack of Von Hippel–Lindau protein (pVHL) can also partly mimic PHD deficiency, since pVHL-deficient mice have a sustained activation of HIF1α and HIF2α, due to disrupted breakdown of HIF proteins. As we observe decreased *Cyp7a1* expression, this may have been the underlying mechanism for enhanced plasma cholesterol in PHD3dko mice. In addition, an increase in fatty acid synthase (FAS) expression could be the cause of the increased triglyceride levels in PHD3dko mice. In cancer, it has been shown that hypoxia can induce FAS expression, possibly via sterol regulatory element-binding protein 1c (SREBP1c). However, the hepatic expression of SREBP1c did not differ between PHD3dko and controls, indicating that the activation of FAS might occur via a different route. One possible pathway could be the induction of Bcl2/adenovirus E1B 19 kDa protein-interacting protein 3 (BNIP3) in hypoxia, leading to the induction of FAS (Lee et al., 2017).

Interestingly, a recent study from Auvinen et al. investigated the relationship between Hb, body mass-index (BMI), and circulating metabolites in two Finnish cohorts: the Northern Finland Birth Cohort 1966 (Rantakallio, 1969; Jarvelin et al., 2004) and Young Finns Study (Raitakari et al., 2008). They found that increased Hb levels were positively associated with BMI (Auvinen et al., 2018). Moreover, Hb levels were positively associated with a unfavorable lipid profile and glucose intolerance, independent of BMI, sex, smoking, and physical activity. However, Mendelian randomization analyses could not establish a causal relationship between Hb and BMI; hence, additional research to address causality is warranted. Nevertheless, experiments in mice that underwent venesection and subsequently showed enhanced Hb levels after 2 weeks also displayed concurrent increase in body weight, circulating glucose and cholesterol levels (Auvinen et al., 2018). The observed increase in Hb with enhanced lipid levels and body weight phenocopied our findings in PHD3dko mice. The mechanistic interplay between Hb, plasma lipid levels and body weight are unfortunately still unchartered. Advancing insights about this relationship might be able to explain our findings in the near future.

The lack of effect on atherogenesis is surprising, considering altered lipid metabolism and the overt phenotypes of the other two isoforms, PHD1 and PHD2 (Rahtu-Korpela et al., 2014; Marsch et al., 2016). Functional effects of PHD3 deficiency in plaque cells may have counteracted the mild, systemic increase in lipids. In addition, cell type-specific effects may have counteracted each other, leaving the net plaque progression unaltered. Cell type-specific knockouts would be needed to convincingly prove this hypothesis. In contrast to our current findings, a study using a short hair-pin approach targeting PHD3 found a beneficial effect on atherogenesis (Liu et al., 2016). The apparent contrasting results may be explained by different methods, i.e., partial KD in adult mice is not similar to complete KO from embryonic stage. In addition, an ApoE knock-out model was used, which manifests in different atherogenic outcome measures compared to LDLrko mice in terms of inflammation, lipid profile, and plaque phenotype (Getz and Reardon, 2016; Oppi et al., 2019).

A third explanation for the lack of effect on atherogenesis may be the potential compensatory upregulation of other PHD isoforms in the arterial wall and other disease-relevant organs following whole-body PHD3 deletion. In our PHD3dko mice, hepatic *Phd1* and *Phd2* mRNA expression was not significantly different compared to controls. According to literature, compensatory upregulation of PHD1 or PHD2 in reaction to PHD3 deficiency and vice versa could occur in other cell types. The extent of these effects is, however, dependent on cell type and the targeted HIF isoform (Appelhoff et al., 2004; Mazzone et al., 2009; Minamishima et al., 2009; Walmsley et al., 2011; Taniguchi et al., 2013). For example, there seems to be an upregulation of PHD1 in MCF7 cells, a breast cancer cell line, treated with PHD3 siRNA (Appelhoff et al., 2004). In contrast, others claim there is no compensatory upregulation of PHD1 or PHD2 in neutrophils isolated from PHD3ko mice (Walmsley et al., 2011). It remains to be studied if this was the case in the arterial wall. Another surprise was that plaque hypoxic content was similar, despite a slight increase in hemoglobin and hematocrit in PHD3dko mice. PHD1 deficiency in atherosclerotic plaques and heart reduced oxygen consumption and tissue hypoxia (Aragones et al., 2008; Marsch et al., 2016). In summary, even though PHD3dko mice are mildly dyslipidemic and polycythemic, PHD3 deficiency does not worsen atherogenesis. These effects are in contrast to effect size and directionality of the other PHD isoforms, suggesting PHD isoform-dependent effects on cardiovascular disease metabolism.

## Data Availability Statement

The raw data supporting the conclusion of this article will be made available by the authors, without undue reservation.

## Ethics Statement

The animal study was reviewed and approved by the Animal Welfare Body, Maastricht University Medical Centre.

## Author Contributions

TT and JS conceived and designed the study. JD, KK, TT, EM, and SH performed the experiments, and analyzed the data. PC generated the mouse model. JD, KK, and JS wrote the main manuscript text. JD, TT, and JS prepared the figures. EF, EB, and PC provided critical input to the manuscript. All authors reviewed and approved the final manuscript.

## Conflict of Interest

The authors declare that the research was conducted in the absence of any commercial or financial relationships that could be construed as a potential conflict of interest.

## Abbreviations

ABCB5/8: ATP-binding cassette subfamily G member 5/8
BMDM: bone marrow-derived macrophages
CD36: cluster of differentiation 36
CKD: chronic kidney disease
CVD: cardiovascular disease
CYP7a1/8b1: cytochrome P450 7a1/8b1
dko: double knockout
EGLN: egl-9 family hypoxia inducible factors
FAS: fatty acid synthase
HCD: high-cholesterol diet
HIF: hypoxia inducible factor
HMGCR: 3-hydroxy-3-methylglutaryl-CoA reductase
H&E: hematoxylin and eosin
IRS-2: insulin receptor substrate 2
LRLR: low-density lipoprotein receptor
LRP1: low-density lipoprotein receptor-related protein 1
PHD: prolyl hydroxylase
PPAR γ: peroxisome proliferator-activated receptor gamma
pVHL: Von Hippel–Lindau protein
SCARB1: scavenger receptor class B type 1
SREBPc1: sterol regulatory element-binding protein
TG: triglycerides.
