# Supplementary figures and images for: Whole-Body Prolyl Hydroxylase Domain (PHD) 3 Deficiency Increased Plasma Lipids and Hematocrit Without Impacting Plaque Size in Low-Density Lipoprotein Receptor Knockout Mice
Source: Front Cell Dev Biol. 2021 May 14;9:664258. doi: 10.3389/fcell.2021.664258 (PMC8160238; doi:10.3389/fcell.2021.664258)

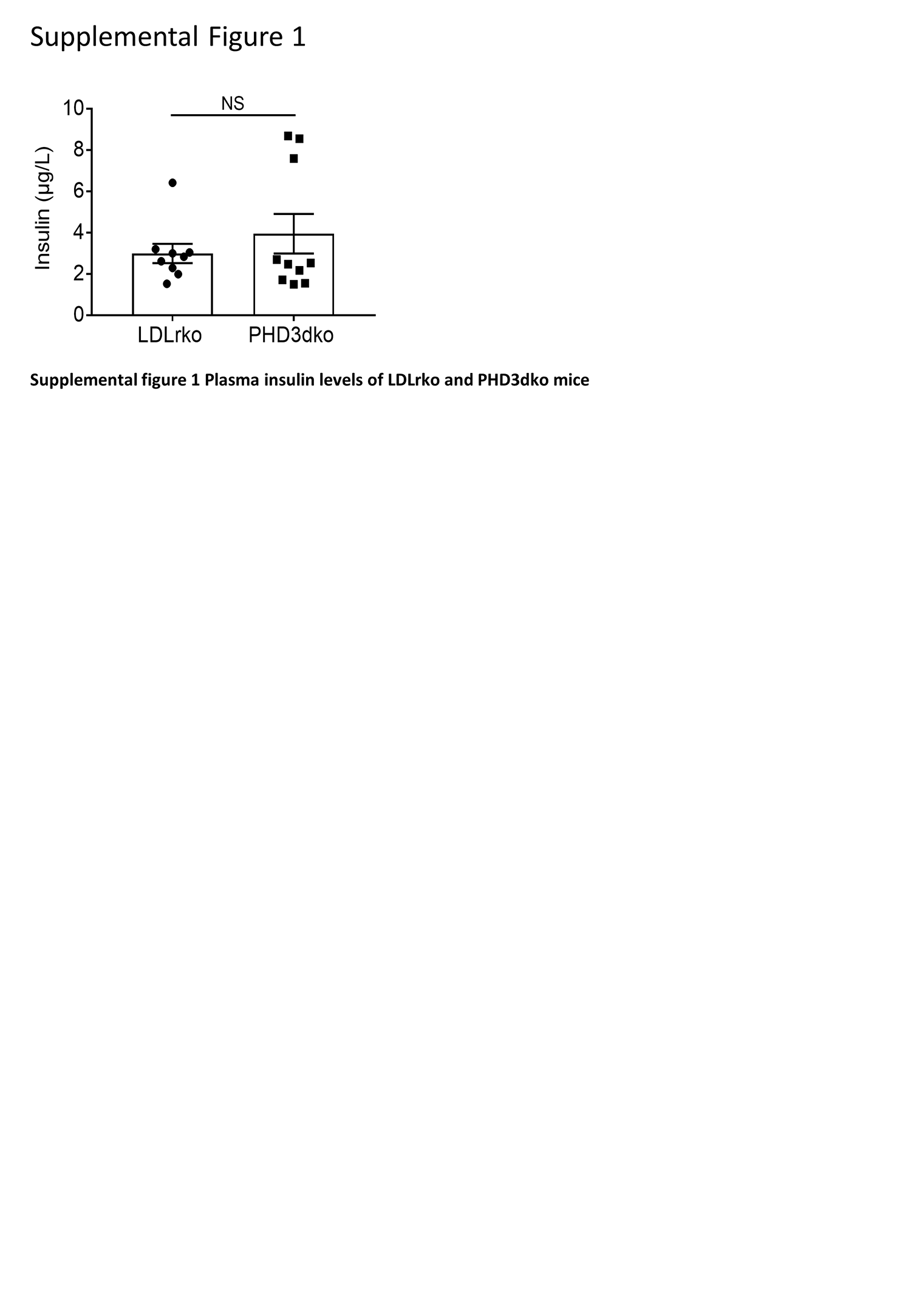

Supplement: Supplementary file 1 [file Image_1.TIF]

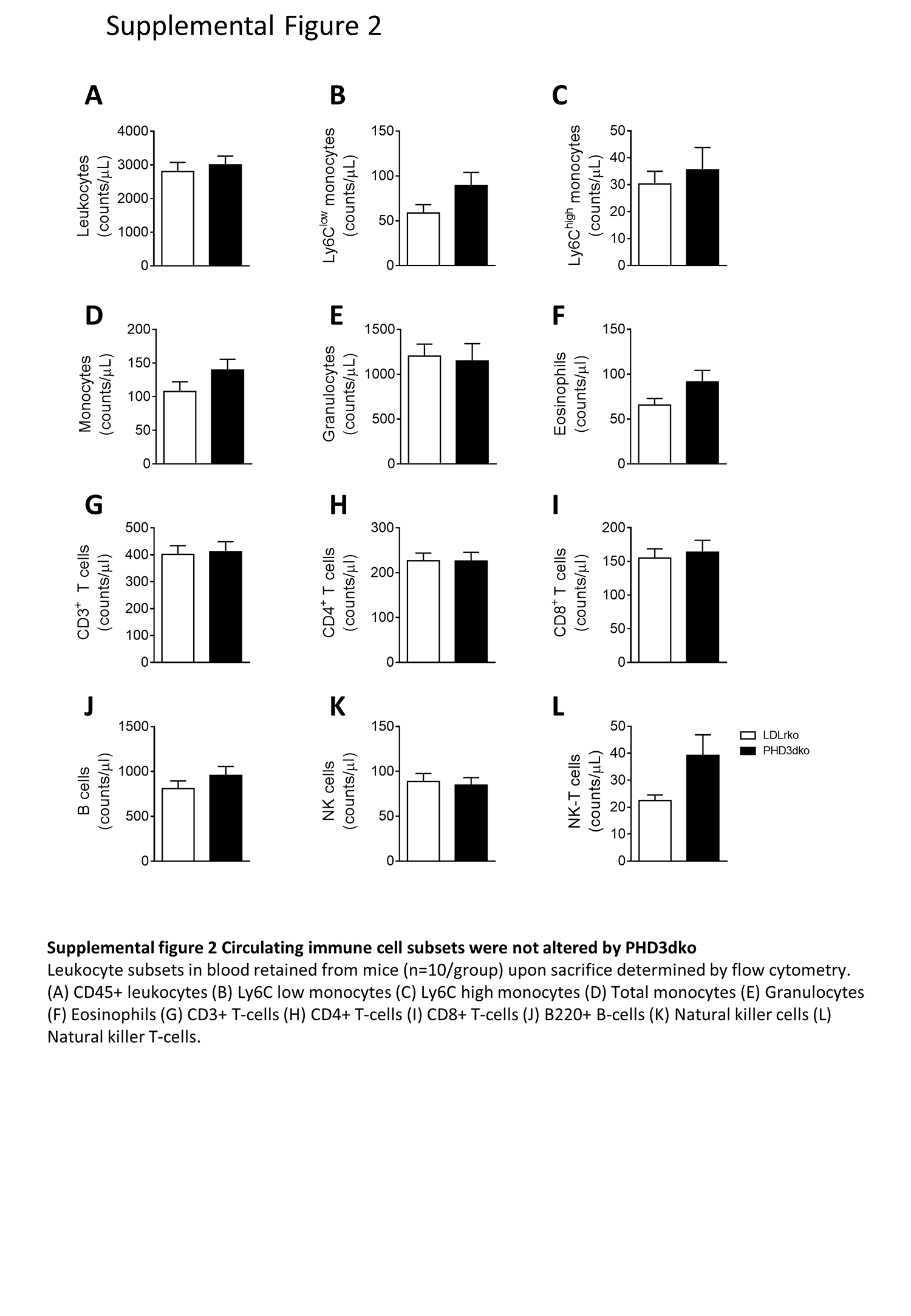

Supplement: Supplementary file 2 [file Image_2.TIF]
